# Comparison of focused cognitive training and portable “brain-games” on functional outcomes for vocational rehabilitation participants

**DOI:** 10.1038/s41598-018-20094-w

**Published:** 2018-01-29

**Authors:** Morris D. Bell, Holly Laws, Brian Pittman, Jason K. Johannesen

**Affiliations:** 1 VA Connecticut Healthcare System, West Haven, CT, USA; 2 Department of Psychiatry, Yale School of Medicine, New Haven, CT, USA

## Abstract

Cognitive remediation performed in a cognitive laboratory was compared with a sham control using portable brain games to study effects on vocational, neurocognitive, and functional outcomes for participants with psychotic disorders in vocational rehabilitation (VR). Seventy-seven participants (61% schizophrenia, 39% other psychosis) in transitional (45.5%) or supported employment (54.5%) were randomly assigned to 6 months of portable cognitive-games (CG) or cognitive remediation (CR) plus a weekly goal-setting group, and evaluated during training, post-training and at 12 months. Overall rates of employment did not differ significantly at 12-month follow-up; however, VR + CG attained employment more rapidly during training. A significant time by condition interaction favored VR + CR on Quality of Life Total Score and Instrumental Functioning over 12 months. Neurocognitive outcomes favored VR + CR, particularly on attention. Training hours related significantly to neurocognitive improvement regardless of condition. No differences were found in training adherence despite portability for VR + CG. Results indicate that VR + CR had significantly greater effect than VR + CG on neurocognition and community functioning, but not on employment outcome. Job attainment rates during the training period revealed a potential advantage for portable training raising new questions concerning how cognitive remediation can be most effectively integrated with VR.

## Introduction

Recent reviews of cognitive remediation (CR) for people with schizophrenia have found moderate effect sizes on neurocognitive outcomes, and there has been growing evidence for generalized benefits to social role functioning when paired with rehabilitation interventions. McGurk *et al*.^1^ performed a meta-analysis of 26 RCTs and found effect sizes of 0.41 for cognitive outcomes and 0.36 for psychosocial functioning; Wykes *et al*.^2^ performed a meta-analysis of 40 studies and reported effect sizes of 0.45 for cognitive improvement and 0.42 for social functioning; and Katsumi *et al*.^3^ recently did an updated literature review and reported similar findings.

Our research group (Wexler & Bell^4,5^) and others (e.g. McGurk *et al*.^6^; Nuechterlein *et al*.^7^) have focused on CR as an augmentation to vocational rehabilitation (VR), reporting favorable employment outcomes. A meta-analysis of 9 studies from 5 different countries^8^ found that participants receiving CR showed 20% higher employment rate, worked more days and earned US $959 more than those who did not receive CR. They concluded that CR plus vocational services enhanced productivity and quality of life. Recently, Lystad *et al*.^9^ reported on a complex study in Norway that compared CR with Cognitive Behavior Therapy (CBT) for participants with schizophrenia spectrum disorders in VR programs over two years. They found that both groups improved on several neurocognitive domains, though results favored CR. Vocational outcomes did not differ by condition, although cognitive improvement in CR uniquely predicted hours worked.

As efforts to integrate CR into clinical and rehabilitation activities continue, it is important to learn as much as possible about its effectiveness across various rehabilitation programs in everyday practice. Wykes and Spaulding^10^ in a seminal paper argue that we need to know about the clinical significance of CR, its impact on functioning and what features of the intervention are most important and for whom.

The present report describes a study designed with some of these aims in mind. Data collection was begun in 2009 and completed in 2015. To our knowledge, it is the first study conducted on individuals with schizophrenia-spectrum illness designed to compare CR in the context of a range of vocational rehabilitation services with an active sham control comprised of portable “brain-games.” The primary aim was to compare a scientifically validated focused cognitive training procedure (Brain Fitness and Insight by Posit Science) performed in a cognitive training laboratory (Cog Lab) to non-specific “brain games” (Brain Age-2) that would serve as a rigorous sham control. We believed that Brain Age-2 was an excellent choice as a sham control because it had face valid exercises of executive function and provided user-friendly feedback in the form of a “Brain Age” score, that indicated progress toward “younger” (better) cognitive performance. However, we believed it was a sham because unlike the targeted training of Posit Science’s auditory (Brain Fitness) and visual (Insight) exercises, the Brain Age-2 had no training that addressed primary sensory processing and had very limited within-exercise adaptations to performance. No CR clinical trial had used such a face valid comparator at the time this study was proposed. Recently, a review of 18 commercial CR programs concluded that Posit Science had by far the strongest scientific evidence of effectiveness, that Brain Age-2 was in the second tier of evidence, while almost all others had either poor evidence or none at all^11^. Thus, it appears that Brain-Age 2 has some scientific support for cognitive benefits, at least in healthy adults^12,13^.

In addition, Brain Age-2 was on a portable, hand-held device (Nintendo DS), which allowed us to ask a second question regarding the impact of portability on adherence. While most previous studies including our own supported the benefits of targeted CR performed on site^14^, Brain Age-2 had the advantage that participants could use it outside of laboratory hours, and thus might train much more. Moreover, because portable training allows users to train at their convenience, it was less likely to conflict with job searching or establishing a new work schedule. At the time of this study, there were no tablet ready versions of Posit Science’s targeted CR exercises. Had there been, we might have focused our study on comparing portable training with Cog Lab training. Although we couldn’t do that comparison, it was still an important question to test whether portability would lead to more training adherence. For these reasons, we hypothesized that (1) scientifically supported CR training would be more effective in improving neurocognitive function than a face valid, sham control, and that these neurocognitive benefits would generalize to superior vocational outcomes and community functioning; and (2) portability of computer training would lead to more overall training activity.

## Results

### Sample Characteristics

Baseline demographic, illness and employment characteristics (Table 1) showed no significant differences between conditions, except for a greater number of participants with schizophrenia and schizoaffective disorder (when combined) for the Posit Science group (72% vs 50%, *p* < 0.05). Most of the participants were receiving SSDI, SSI or VA service-connected benefits (89.6%) and hadn’t worked on average in almost 11 years. Average earnings in the last 30 days was about $25.00. At baseline 30 (39%) had not begun their vocational rehabilitation service at intake but were in process; 24 (31%) had just begun working in Incentive Therapy; 15 (19.5%) were in SE though none were currently working; 6 (8%) had begun working in Compensated Work Therapy at the VA, and 2 (2.6%) were working casual jobs a few hours a week. All participants were eventually enrolled in some form of VR with slightly more than half in SE (54.5%). There was no difference between conditions in type of VR services used (Table 1).

**Table 1:** Baseline Characteristics.

Arm/Group Title	VR + CG (% within condition)	VR + CR (% within condition)	Total
Total Baseline Participants	38	39	77
Age in years Mean (SD)	52.87 (10.90)	49.54 (12.90)	51.18 (12.00)
Female	5 (13%)	3 (8%)	8 (10.4%)
Male	33 (87%)	36 (92%)	69 (89.6%)
**Ethnicity (NIH/OMB)**			
Hispanic or Latino	2 (5%)	1 (3%)	3 (4%)
Not Hispanic or Latino	36 (95%)	36 (92%)	72 (93.5%)
Unknown or Not Reported	0	2 (5%)	2 (2.5%)
**Race (NIH/OMB)**			
American or Alaska Native	1 (2.7%)	1 (2.6)	2 (2.5%)
Black or African American	17 (44.7%)	19 (48.7%)	36 (46.8%)
White	18 (47.3%)	16 (41.0%)	34 (44.2%)
Unknown or Not Reported	2 (5.3%)	3 (7.7%)	5 (6.5%)
**Marital Status**			
Married	3 (8%)	3 (7.7%)	6 (7.8%)
Never Married	18 (47.4%)	24 (61.5%)	42 (54.5%)
Divorced/Widowed	17 (44.7%)	12 (30.8%)	29 (37.7%)
**Axis 1 Primary Diagnosis**			
Schizophrenia	14 (36.8%)	18 (46.2)	32 (41.6%)
Schizoaffective Disorder	5 (13.2%)	10 (25.6%)	15 (19.4)
Other Disorder	19 (50%)	11 (28.2%)	30 (39.0%)
Educ. years Mean (SD)	12.51 (1.41)	12.49 (2.27)	12.50 (1.88)
Disability (SS or VA)	33 (86.8%)	36 (92.3%)	69 (89.6%)
Mon. Since Last Em-ployment Mean (SD)	122.11 (105.90)	140.82 (137.78)	131.20 (121.88)
Dollars earned in Last 30 Days Mean (SD)	47.54 (117.25)	3.69 (17.32)	25.04 (85.06)
Full Time Past Employment >1 Yes	33 (86.8%)	27 (69.2%)	60 (78.0%)
GAF Mean (SD)	49.92 (10.941)	46.36 (9.152)	48.12 (10.167)
PANSS Total Mean (SD)	56.03 (11.47)	56.26 (11.06)	56.14 (11.19)
QLS Total Mean (SD)	66.84 (18.28)	64.64 (15.65)	65.73 (16.92)
**Type of Voc. Rehab. Program**			
Transitional Work	18 (47.4%)	17 (43.6%)	35 (45.5%)
Supported Employment	20 (52.6%)	22 (56.4%)	42 (54.5%)

No statistically significant differences were found between conditions. (Abbreviations: Educ = Education; SS = Social Security Disability Income or SSI = Supplemental Security Income, VA = Service Connected Disability; Mon = Months; PANSS = Positive and Negative Symptom Scale; GAF = Global Assessment of Function); Voc = Vocational).

The 13 participants from the CMHC sample did not differ on PANSS scores at baseline, but they did differ significantly on QoL scores (VA mean = 67.9 (17.07); CMHC mean = 54.92 (11.45); t (75) = 2.62, p < 0.01). However, there was no significant difference between the two conditions regarding what site they came from (Chi Sq = 0.74, p = ns). The 13 participants from CMHC represents only 16.8% of the whole sample, and when site was added as a covariate in subsequent analyses, it had no effect on findings.

### Retention and Adherence

Retention was somewhat better for VR + CR at 6- (87% vs 74%) and 12- month follow-up (74% vs 71%) but not statistically different (Consort Flow diagram in Supplementary Material). Adherence to treatment did not differ by condition (F(1,74) = 1.19, p = 0.29). A few participants spent a lot of time on Brain Age-2, influencing a higher mean number of training hours in the VR + CG condition but with a very large standard deviation and range (VR + CG M = 63.5, SD = 65.12, Range 0–193.00). When categorized into 3 levels of training (less than 14 hours; 14 to 60 hours; more than 60 hours), the conditions also did not differ significantly (Χ^2^_(2)_ = 3.87, p = 0.15). While both conditions showed generally good attendance at the weekly goal-setting group, VR + CR participation (mean = 19.27 (7.95) groups out of 26 weeks) was significantly greater than that of VR + CG (mean = 14.78 (9.90), t(72) = 2.15, p < 0.05).

### Employment Outcome

**Figure 1:**
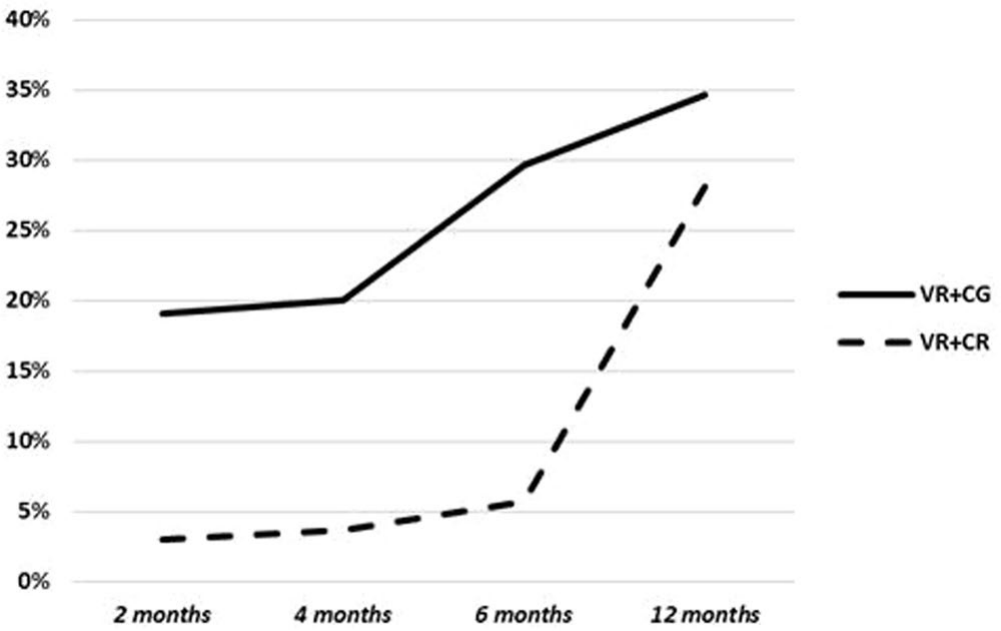
Employment rates over time by condition.

Conditions did not differ by competitive employment rate at 12-month follow-up (VR + CG = 9/38, VR + CR = 9/36; Χ^2^_(1)_ = 0.02, *p* = 0.89). However, non-parametric mixed-effects model of employment attainment at 2, 4, 6 and 12 months revealed significant condition (Χ^2^_(1)_. = 4.73, *p* < 0.03) and time (Χ^2^_(3)_ = 13.94, *p* < 0.01) effects favoring VR + CG, but no time-by-condition interaction (Χ^2^_(1)_ = 4.25, *p* = 0.24). This analysis indicated that VR + CG led to significantly earlier job attainment during the 6-month active training period, but those receiving VR + CR caught up over the 6-month follow-up period (Figure 1).

Hours engaged in training was not significantly associated with employment attainment (*b* = 0.003, *se* = 0.005, *p* = 0.591, *OR* = 1.003). Non-parametric analysis revealed significant differences in employment hours and earnings at 6 months favoring VR + CG (Table 2), but no difference at 12 months. Mixed-models analyses of employment hours using all time points (2 months, 4 months, 6 months, 12 months), showed a condition effect favoring VR + CG for employment hours (*df* = 1, ATS = 6.21, *p* < 0.013), but no time or time-by-condition interaction. There was a significant decline in overall productive hours across groups by the end of 12 months (time effect: *df* = 2.62, ATS = 5.56, *p* < 0.001), but no time-by-condition interaction (Supplements S2 & S3.) The decline in hours over time was mostly due to the 6-month transitional work program, which ended around the 6-month assessment. Since most recipients of this service didn’t get employed, their productive hours declined sharply by 12 months. Of note, 41.2% of participants receiving Supported Employment (SE) attained employment over 12 months compared with 16.7% of those in transitional employment a significant difference (Χ^2^_(1)_ = 3.94, *p* < 0.05).

**Table 2:** Employment Outcomes.

Variable	VR + CG						VR + CR						
	N	Mean	Std Dev	Std Error	Min	Max	N	Mean	Std Dev	Std Error	Min	Max	NonPar
Competitive Hours 6	29	51.1	126.6	23.5	0	650	36	19.1	85.7	14.3	0	480	0.02
Competitive Hours 12	30	101.4	234.5	42.8	0	1010	36	60	194.4	32.4	0	960	0.10
Productive Hours 6	29	344.1	476.4	88.5	0	2330	36	244.1	288.6	48.1	0	840	0.37
Productive Hours 12	30	443.3	569.7	104	0	2330	36	324.9	347.3	57.9	0	1022	0.61
Competitive Earnings 6	29	459.2	1089.8	202.4	0	5363	36	142.2	628.4	104.7	0	3480	0.02
Competitive earnings 12	30	941.8	2038.7	372.2	0	8333	36	438.10	2321.93	381.7	0	7037	0.12
All Earnings 6	29	1483.2	1886.7	350.3	0	7680	36	1005.8	1309.4	218.2	0	4555	0.28
All Earnings 12	30	2106.7	3622.6	661.4	0	17904	36	1513.0	2059.2	343.2	0	7946	0.82

^*^Wilcoxon Test (Abbreviations: N = number; Std Dev = Standard Deviation; Std Error = Standard Error; Min and Max = Minimum and Maximum Non Par = Non-parametric p value).

### *Neurocognitive Outcomes (*Table 3)

Mixed-models indicated no main effect for condition on baseline neurocognitive measures (Tables 3 and 4). A significant time-by-condition interaction in the Attention domain (*p* = 0.034) indicated significant increases in Attention over 12 months (*p* = 0.002) in VR + CR, and no appreciable change from baseline in VR + CG (*p* > 0.50; Supplement S4). No other between-condition effects were detected on neurocognitive outcomes.

**Table 3:** Descriptive Statistics^a^ for Quality of Life (QLS scale), Neurocognitive, and Employment Outcomes by Treatment Condition.

	Baseline		2 months		4 months		6 months		12 months	
	VR + CR	VR + CG	VR + CR	VR + CG	VR + CR	VR + CG	VR + CR	VR + CG	VR + CR	VR + CG
	*N* = 39	*N* = 38	*N* = 33	*N* = 22	*N* = 27	*N* = 20	*N* = 34	*N* = 26	*N* = 29	*N* = 27
Instrumental	6.56	8.03	9.21	12.72	7.93	9.35	6.47	7.04	7.38	4.67
	*7.88*	*8.27*	*7.98*	*8.37*	*7.73*	*8.07*	*7.75*	*8.11*	*7.15*	*7.25*
Interpersonal	22.28	22.34	22.64	23.95	22.70	24.30	23.35	22.46	21.86	21.07
	*7.90*	*10.78*	*8.66*	*9.85*	*8.30*	*8.94*	*8.87*	*9.34*	*8.53*	*7.25*
Intrapsychic	28.62	28.47	28.45	30.45	27.56	30.40	28.62	28.92	28.17	26.48
	*6.43*	*6.18*	*5.80*	*7.09*	*7.39*	*5.18*	*5.48*	*6.77*	*5.97*	*6.78*
Objects/Activities	7.18	8.00	7.24	8.41	7.04	8.45	7.71	8.08	7.48	8.48
	*1.67*	*1.96*	*2.19*	*1.22*	*2.62*	*1.57*	*2.13*	*1.62*	*1.86*	*1.45*
Total QLS Score	64.64	66.84	67.55	75.55	65.22	72.50	66.15	66.50	64.90	60.70
	*15.65*	*18.28*	*16.16*	*21.95*	*18.64*	*15.41*	*14.72*	*19.09*	*17.00*	*17.98*
Processing Speed	38.26	35.96					37.62	37.27	38.45	37.85
	*10.13*	*11.74*					*9.64*	*13.22*	*12.03*	*10.66*
Attention	36.03	36.89					38.74	36.92	41.24	36.11
	*11.49*	*10.21*					*11.97*	*10.16*	*10.60*	*12.29*
Working Memory	35.23	34.13					35.79	37.54	35.69	37.30
	*9.26*	*13.58*					*10.47*	*13.67*	*11.34*	*12.78*
Verbal Learning	39.21	39.03					34.79	37.19	36.08	37.63
	*8.01*	*5.56*					*6.86*	*7.21*	*8.19*	*8.71*
Visual Learning	36.08	41.11					38.53	44.19	39.14	44.48
	*11.00*	*13.15*					*13.30*	*15.24*	*11.87*	*12.78*
Problem Solving	40.31	40.42					40.67	39.45	40.28	43.04
	*8.01*	*8.44*					*9.43*	*14.60*	*9.09*	*9.76*
Global Cog	29.03	29.89					29.89	31.99	31.66	32.07
	*9.09*	*11.77*					*10.33*	*13.46*	*11.30*	*12.68*
Total Training Hrs	VR + CR: *M* = 49.09 *SD* = 35.57, *Range* 0–118.83					VR + CG: M = 63.46, *SD* = 65.16 *(Range* 0–193.00*)*				
Ever Employed^*b*^	VR + CR: 11 of 36 participants (30.6%) VR + CG: 10 of 30 participants (33.3%)									

Means for all outcomes, SDs are directly below each mean in italics; ^*b*^Participants who obtained Employment anytime during the study.

**Table 4:** Mixed Model Coefficients and Associated Standard Errors Testing Effects of VR + CR Versus VR + CG on Change in Neurocognitive Outcomes

	Processing Speed		Attention		Working Memory		Verbal Learning		Visual Learning		Problem Solving		Global Cognitive Index	
	Est. *(SE)*	*p*	Est. *(SE)*	*p*	Est. *(SE)*	*p*	Est. *(SE)*	*p*	Est. *(SE)*	*p*	Est. *(SE)*	*p*	Est. *(SE)*	*p*
Intercept	35.87 *(1.74)*	<0.001	36.05 *(1.63)*	<0.001	34.11 *(1.76)*	<0.001	38.48 *(1.12)*	<0.001	40.90 *(1.96)*	<0.001	39.56 *(1.54)*	<0.001	29.44 *(1.69)*	<0.001
Condition	2.34 *(2.45)*	0.342	0.45 *(2.30)*	0.841	1.63 *(2.48)*	0.514	−0.28 *(1.57)*	0.857	−3.88 *(2.75)*	0.162	1.03 *(2.16)*	0.633	−0.11 *(2.38)*	0.965
Time	−0.48 *(1.37)*	0.729	0.004 *(2.30)*	0.846	0.58 *(1.54)*	0.703	−2.36 *(1.32)*	0.077	1.36 *(1.71)*	0.431	1.51 *(1.10)*	0.175	−0.16 *(1.00)*	0.874
Condition*Time	0.47 *(1.85)*	0.799	4.45 *(2.07)*	0.034	0.52 *(2.09)*	0.806	0.18 *(1.79)*	0.919	−0.13 *(2.32)*	0.957	−1.42 *(1.48)*	0.345	1.85 *(1.35)*	0.176
Training Hours	0.17 *(23)*	0.480	0.71 *(0.22)*	0.002	0.42 *(0.24)*	0.084	−2.36 *(1.32)*	0.077	0.63 *(0.26)*	0.019	0.16 *(0.21)*	0.433	0.65 *(0.23)*	0.006
Training Hours*Time	0.34 *(0.17)*	0.059	0.03 *(0.20)*	0.896	0.52 *(0.20)*	0.010	0.06 *(0.17)*	0.702	−0.20 *(0.22)*	0.357	0.02 *(0.14)*	0.900	0.20 *(0*.*13)*	0.116

*Note:* Condition had a value of 1 for VR + CR and a value of 0 for the VR + CG condition. A 1 unit change in Time reflected change over the entire study period of 12 months. The Training Hours variable was divided by 10 for meaningful coefficient interpretation (a 1 unit change per 10 hours of training), and mean-centered to retain an interpretable intercept value.

Additional analyses support the relationship between cognitive training activity and cognitive outcomes across conditions and time. Hours of cognitive training by time predicted change in Working Memory (*p* = 0.010) and Processing Speed (trend level; *p* = 0.059) over 12-month. There were also significant time effects, unrelated to hours of training, for Attention (p = 0.002) and Visual Learning (p = 0.019), and for Global Cognitive Index (p = 0.006). Follow-up analysis of simple slopes indicated that improvements in Working Memory were obtained for individuals who trained 40 hours or more across the treatment period. Three-way interactions between condition, training hours, and time were non-significant.

### *Quality of Life* (*QLS) Outcomes* (Table 3)

Mixed-models indicated no difference in baseline between conditions (Tables 3 and 5). Significant time-by-condition interactions for Total QLS (*p* < 0.02) and Instrumental Function (*p* < 0.01; Supplement S5) indicated that these QLS measures remained stable over time in VR + CR (*p* < 0.50) but declined significantly for VR + CG (QLS Total *p* < 0.01 and Instrumental Function *p* < 0.001). Training hours were associated with Instrumental Function ratings at the trend level (*p* = 0.053).

**Table 5:** Mixed Model Coefficients and Associated Standard Errors Testing Effects of VR + CR vs VR + CG on Change in Quality of Life Outcomes

	Instrumental		Interpersonal		Intrapsychic		Objects/Activities		Total Quality of Life Score	
	Est. *(SE)*	*p*	Est. *(SE)*	*p*	Est. *(SE)*	*p*	Est. *(SE)*	*p*	Est. *(SE)*	*p*
Intercept	9.75 *(1.19)*	<0.001	22.74 *(1.44)*	<0.001	29.08 *(0.97)*	<0.001	7.91 *(0.28)*	<0.001	68.72 *(2.69)*	<0.001
Condition	−1.87 *(1.63)*	0.254	−0.25 *(2.01)*	0.901	*−0.35* *(1.34)*	0.795	−0.60 *(0.39)*	0.123	−2.61 *(3.76)*	0.490
Time	−6.53 *(1.67)*	<0.001	−1.12 *(1.38)*	0.422	−2.50 *(1.17)*	0.036	0.35 *(0.32)*	0.270	−9.00 *(2.87)*	0.003
Condition*Time	6.17 *(2.25)*	0.008	1.20 *(1.86)*	0.522	2.15 *(1.57)*	0.174	−0.06 *(0.43)*	0.892	9.37 *(3.89)*	0.018
Training Hours	*0.10* *(0.16)*	0.536	*0.02* *(0.19)*	0.928	*0.30* *(0.13Tab)*	0.021	*0.09* *(0.04)*	0.017	*0.41* *(0.36)*	0.258
Training Hours*Time	0.42 *(0.21)*	0.053	0.12 *(0.18)*	0.515	−0.08 *(0.15)*	0.602	−0.03 *(0.04)*	0.415	0.56 *(0.37)*	0.133

*Note:* Condition had a value of 1 for VR + CR and a value of 0 for the active control VR + CG condition. A 1 unit change in Time reflected change over the entire study period of 12 months. The Training Hours variable was divided by 10 for meaningful coefficient interpretation (a 1 unit change per 10 hours of training), and mean-centered to retain an interpretable intercept value.

## Discussion

To our knowledge this is the first report comparing focused cognitive remediation in a “Cog Lab” to a sham control of cognitive games on a mobile device in the context of vocational services. Employment outcomes by 12 months in this chronically unemployed and disabled psychiatric sample do not indicate that either condition was superior. There may have been some advantage to having a portable device during the 6-month training phase as suggested by more rapid rate of employment and more hours of competitive work in the VR + CG condition. Furthermore, an upshift in employment in VR + CR following completion of cognitive training, reaching levels equivalent to VR + CG by 12 months, supports the interpretation that participants in this condition only fully engaged in job searching after completing the 6-month curriculum of cognitive training. Since hours spent training did not differ by condition and appeared unrelated to attaining employment by 12 months, it remains possible that coming into the “Cog Lab” rather than using a portable training system may have had an opportunity cost in terms of employment.

In a previous study that compared enhanced SE with 12-month cognitive training along with a social information processing group to enhanced SE alone^5^, we found that although the training condition led to better vocational outcomes at two-year follow-up, those in the “Cog Lab” took longer to obtain a job. The findings of overall superior employment outcome with cognitive training in that study may have been observed due to a longer treatment and follow-up period, which provided more time for the benefits of cognitive training to generalize to vocational function. Moreover, the enhancement of SE included an initial period of paid on-the-job training so that almost everyone began work within 3 months and had the benefit of synergy between cognitive training and work activity. McGurk and colleagues^1,6^ have similarly reported better vocational outcomes using cognitive training but all participants were engaged in SE. Unsurprisingly, in our study, what made the greatest difference in employment was whether participants were enrolled in SE. Those in SE had superior employment rates to those in transitional work programs, regardless of cognitive training condition.

Regarding neurocognitive outcomes, conditions did not differ on MATRICs global score, but VR + CR was superior to VR + CG in the Attention domain. This suggests that despite little overall improvement by either training method, there may have been some specific advantage for the Posit Science training over Brain Age-2 in this domain. We have long maintained that cognitive training produces the greatest benefits when performed in the context of other activating rehabilitation activities. Although, some participants obtained work in our study, most were looking for work or only working a few hours in transitional employment and were otherwise inactive. Cognitive training may be most effective when there are opportunities to generalize and reinforce its lessons through other rehabilitation activity. Nevertheless, we did detect a relationship between amount of training across condition and improvement on MATRICS Working Memory score, suggesting a dose response relationship regardless of the training method. Such findings are unlikely related to practice-effects because the period between assessments is large (6 months) and the MATRICs assessments were selected because they were ones that showed only small practice- effects at much shorter intervals.

Findings for community function as measured by QLS suggest that participants in the VR + CR experienced functional benefits that extended beyond the active intervention period, while those in VR + CG showed consistent decline from baseline over active and follow-up periods. Everyone began the study when they were beginning some type of vocational service, but after initial efforts it appears that the VR + CG group began to be less engaged in productive roles, despite a few more individuals obtaining employment. It may be that the systematic training that led to improvements in attention encouraged VR + CR participants to stay engaged in productive activity, even though it took them longer to get employed. It is also possible that a regular schedule evolved around “Cog Lab” training that created new opportunities to engage in community activity. Participating in more groups than VR + CR may also have increased their capacity for engagement, especially since goal setting included job searching and other forms of productive activity (such as helping family members). Although we do not know the mechanism for the relationship between VR + CR and QLS findings, it is a signal that cognitive remediation with goal-setting groups may be useful in augmenting rehabilitation efforts.

We hypothesized that a mobile device might lead to more training; however, both conditions showed good engagement in cognitive training with no significant difference in overall training hours. A few participants did use their portable device a great deal more than those who were limited to training during lab hours, but this was uncommon. There was, however, a significant difference in group attendance, suggesting that VR + CR participants may have been more likely to attend group since they were already attending “Cog Lab.” It is also possible that since VR + CG participants obtained employment sooner, going to groups conflicted with work activity. Now that many cognitive training programs including Posit Science’s Brain HQ are available on tablets, the option of portability might provide both the benefits of more focused training and times of training more compatible with individual schedules. It remains unknown, however, whether the assistance of a “Cog Lab” monitor and the added social experience of being with others in groups and in the “Cog Lab” offers unique benefits.

Limitations to these conclusions include the lack of a no-treatment control condition, the powerful disincentives to competitive employment in a sample where 90% are receiving disability payments, and the relatively small size of the sample. Only about half of the participants received SE, and since SE had the greatest impact on vocational outcomes its effects may have overshadowed other influences.

While we observed an advantage of laboratory-based over portable device training on cognitive outcomes, these gains did not appear to translate immediately to work outcome. Indeed, given that “Cog Lab” attendance may have impeded job seeking or attainment, the advantages of cognitive gains may come at the cost to other opportunities. Therefore, for those who identify work as a primary goal, we might recommend starting the job search first and offering cognitive training once the client is stable on the job and a need for cognitive remediation has been identified based on job performance or other client goals. Consistent with the supported employment model, the principles of “place then train” could apply appropriately to cognitive remediation interventions targeting vocational outcome.

## Method

### Participants

Seventy-seven participants including 64 Veterans at VA Connecticut Healthcare System (VACHS) and 13 non-veterans at Connecticut Mental Health Center (CMHC) with chronic and disabling psychotic illness (61% schizophrenia, 39% other psychosis) in transitional work services (45.5%) or supported employment (54.5) were included in the study. This was originally intended to be for Veterans only, but the study was modified in its third year to include non-Veterans from CMHC to meet recruitment goals. Inclusion criteria included willingness to engage in vocational rehabilitation services of some kind and presence of disabling psychotic illness. Additionally, vocational programs had some admission requirements including demonstrated need for services, referral from clinicians, and an interest in work rehabilitation. All participants were unemployed, though a few were earning money through informal work. Co-morbid substance use disorder was an exclusion with less than 30 days of abstinence. Known neurological disease, uncorrected sensory impairment, intellectual deficiency or premorbid IQ estimate under 70 were additional exclusion criteria.

### Measures

#### Clinical, Quality of Life and Vocational Assessments

Structured Clinical Interview for DSM IV (SCID)^15^ was used for diagnostic evaluation at baseline. Positive and Negative Syndrome Scale (PANSS)^16^ was used for symptom assessment at baseline and quality of life was measured using Quality of Life Scale interviews (QLS)^17^. QLS has five subscales: Intrapsychic, Interpersonal, Instrumental Function and Objects and Activities. Our raters achieve good to excellent inter-rater reliabilities on all subscales on both instruments^18,19^. QLS was administered at baseline, 2-month, 4-month, 6-month and 12-month follow-ups. Vocational data was recorded at the same observation points and included job attainment, competitive hours worked and money earned, transitional hours worked and money earned, and volunteer work. These data were collected from the vocational program records which were available for Incentive Work Therapy (an in-house work placement that pays half minimum wage and has reduced expectations), Compensated Work Therapy (paid work placements through contracts) and SE. The very few participants who worked independently outside of SE reported their earnings at 6 and 12-month follow-up. Informal income was included in competitive earnings. Types of jobs obtained and reasons for termination were also recorded for qualitative purposes, but are not included in this report.

### Neurocognitive Assessments

Premorbid intellectual functioning was assessed at baseline using the Wechsler Test of Adult Reading^20^. The MATRICS battery^21^ was used to assess changes in cognitive performance over time from baseline to 6-month and 12-month follow-up. Performance was evaluated based on age- and gender-corrected standard scores in domains of Processing Speed, Attention/Vigilance, Working Memory, Verbal Learning, Visual Learning, Reasoning and Problem Solving, and a global neurocognitive composite of these scores.

### Procedures

All procedures were approved by the Institutional Review Boards at VA VACHS and Yale and were carried out in accordance with the relevant guidelines and regulations. Recruitment was done by clinician referral and by publicly displayed flyers. Following informed consent, participants had baseline assessments and if found eligible were randomized in blocks of 6 (3 to Cognitive Games (VR + CG) and 3 to laboratory-based Cognitive Remediation (VR + CR) in a 1:1 ratio by a statistician unrelated to the study. Blocks of 3 were used to ensure nearly equal numbers in both conditions while keeping assessors blind to condition. VR services were unrelated to randomization; individuals had free access to all available VR services. Trained master’s level or Ph.D. research staff blind to condition performed assessments. Training began in the “Cog Lab” located at the Learning Based Recovery Center (LBRC) at VACHS, but VR + CG participants were given the option of subsequently training at home following 3 sessions of instruction about how to use the portable devices. Goal-setting groups occurred weekly at the LBRC.

### Vocational Rehabilitation (VR) Programs

VACHS and CMHC had a range of work services that included transitional programs and Individual Placement and Support^22^ SE programs. IPS programs at both institutions were independently evaluated for fidelity and were in the acceptable range. The transitional programs included paid placement in work therapy at VACHS or at a psychosocial clubhouse with staff support and guidance for job seeking.

### Interventions

#### VR + CG (Nintendo Brain Age-2)

Participants received a hand-held Nintendo DS with “Brain Age-2” training games. They could train as much as they wished, but staff recommended training 1 hour each day, five days per week. They were also asked to do the “Brain-Age” assessment with each day of exercise. They could use the device at our “Cog Lab” or take it home with a $30 deposit. Daily training activity was logged by the Nintendo device and transferred to the research record at weekly goal-setting meetings.

#### VR + CR (Posit Science Brain Fitness and Insight)

Participants performed exercises that targeted auditory and visual discrimination and memory. This training occurred in the “Cog Lab”, available 7 hours a day, 5 days a week, following training schedules created around work and other appointments. A laboratory monitor provided a friendly and encouraging environment, recorded training activity, and gave technical assistance as needed. After a short time, most participants could work through the exercises with minimal assistance. If a participant became sleepy or frustrated during a task, the monitor suggested taking a break before continuing. The monitor, however, did not coach participants in strategies to improve performance. Participants were encouraged to attend 5 laboratory sessions per week, with exercise selection and duration of sessions determined by software. Training progress was logged into a laboratory manual and reviewed with participants at each session. The “Cog Lab” did not include any specific protocol for encouraging socializing or for “buddying up” among the participants; the environment was friendly but business-like, and each person trained on their own.

#### Goal-setting Group

All participants attended the same weekly group regardless of condition assignment. There was no discussion of cognitive training condition in these groups. These groups focused on setting VR goals, reviewing achievements, problem-solving barriers to achievement, and providing encouragement and support while celebrating successes. These goal setting groups were led by a vocational rehabilitation specialist. In addition, ball-draws for rewards for cognitive training participation (see Compensation) occurred in the group and was a source of light-hearted fun.

### Compensation

Participants were compensated for participating in each assessment and could earn $235 in total for completing all assessments. There was no fixed reimbursement for training activity. To avoid giving participants the impression that CR was a paid job, they were not paid a specific hourly rate for training. Rather, training was incentivized using contingency management^23^, a procedure commonly used to encourage drug abstinence. This procedure involved drawing for monetary prizes like a lottery based on attendance. Draws were earned following blocks of 5 training sessions, with the number of draws increasing progressively as training advanced. Each draw had a 2-out-of-5 chance of a monetary award of $1 (1/5 odds) to $25 (1/50 odds).

### Statistical Analyses

Employment outcomes were highly skewed and were analyzed using the nonparametric approach for repeated measures^24^. Data were ranked, then fitted using a mixed-effects model with an unstructured variance-covariance matrix and p-values adjusted for ANOVA-type statistics (ATS). Predictors included condition (VR + CG vs. VR + CR), time, and group-by-time. Employment rates were analyzed across time using a generalized linear model with a logit link function and random subject effects and included the same predictors as above. A logistic regression analysis tested differences between conditions in employment status at study end. Number of training hours was tested as a covariate.

Cognitive and QLS outcomes were analyzed using mixed-effects models that account for the correlation of repeated measures within participants while allowing for inclusion of cases with incomplete or missing data. Models included a random intercept and had condition, time, and group-by-time as predictors. MATRICS measures were collected at 3 time points (baseline, 6, and 12 months). QLS and employment, volunteer and transitional work hours and earnings were collected at 5 time points (baseline, 2, 4, 6, and 12 months). All tests-were two tailed with alpha set at 0.05.

## Electronic supplementary material

CONSORT Diagram and figures

CONSORT Checklist
