# Supplementary material for: Comparison of focused cognitive training and portable “brain-games” on functional outcomes for vocational rehabilitation participants
Source: Sci Rep. 2018 Jan 29;8:1779. doi: 10.1038/s41598-018-20094-w (PMC5789082; doi:10.1038/s41598-018-20094-w)
Supplement: Supplementary file 1 — CONSORT Diagram and figures [file 41598_2018_20094_MOESM1_ESM.pdf]

Comparison of focused cognitive training and portable “brain-games” on functional outcomes for vocational rehabilitation participants

Morris D. Bell[1, 2], Holly Laws[1, 2], Brian Pittman[2], Jason K. Johannesen[1, 2]

[1] VA Connecticut Healthcare System

[2] Yale School of Medicine, Department of Psychiatry

S1. Consort Flow Diagram Intervention

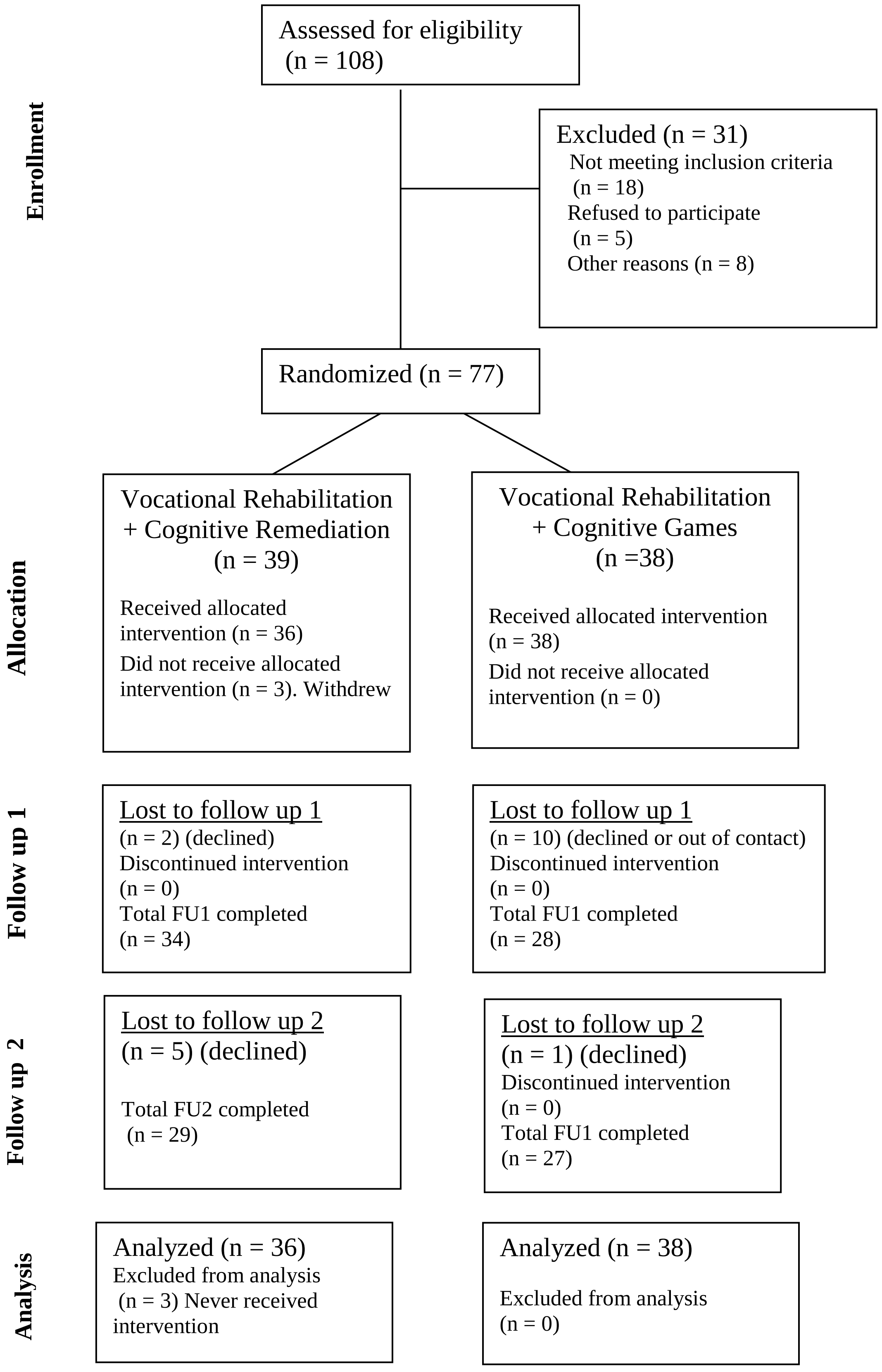

S2. Competitive Hours

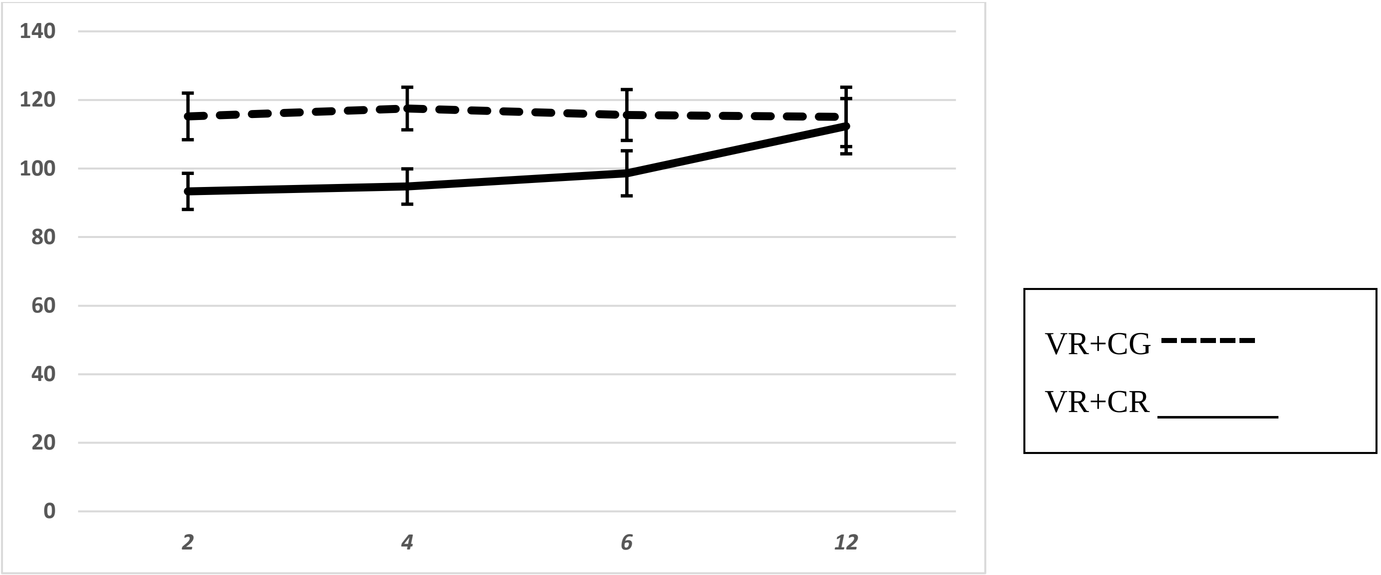

S3. All Productive Hours

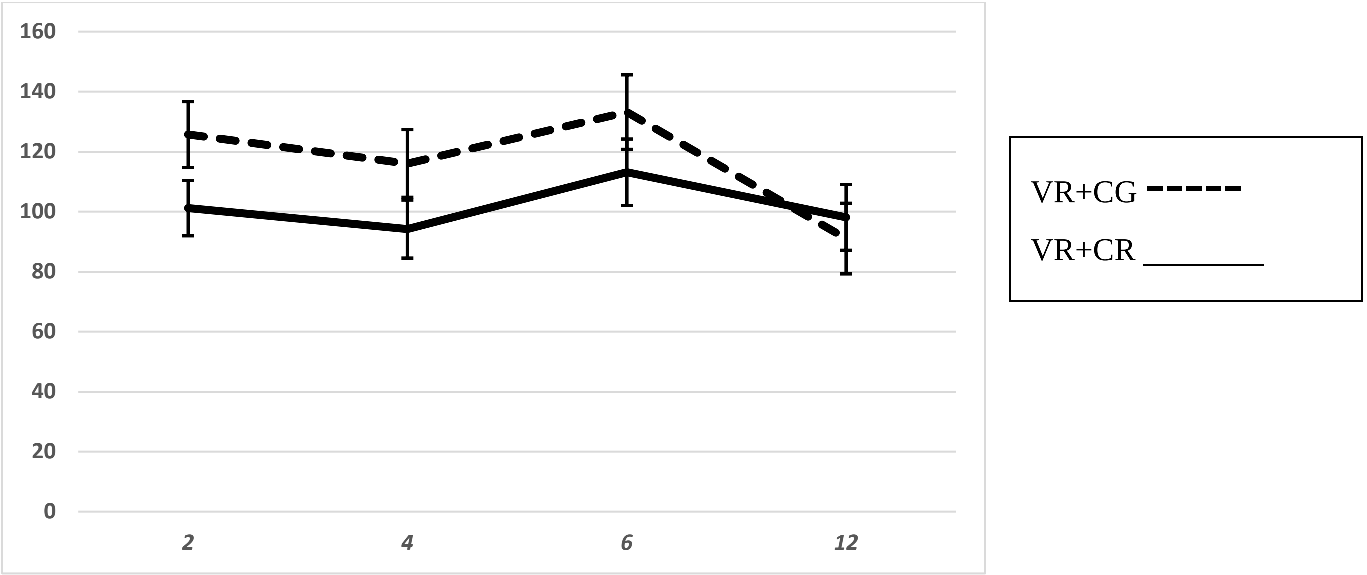

S4. Attention by Time and Condition

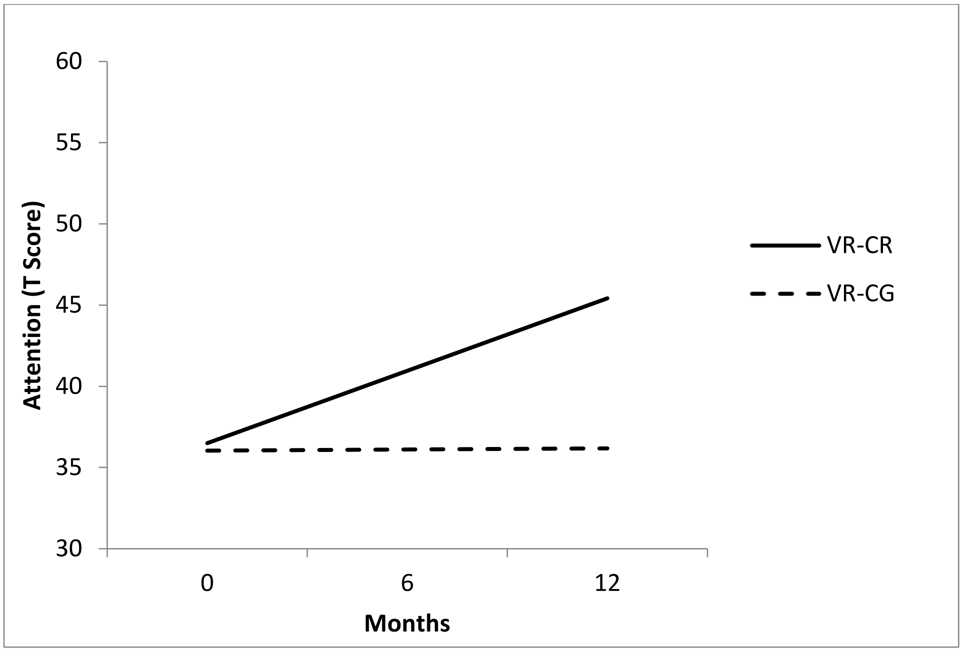

Attention improved significantly more in the Vocational Rehabilitation + Cognitive Remediation (VR-CR; solid lines) compared with the active control Vocational Rehabilitation + Cognitive Games condition (VR-CG; dashed lines). Training hours fixed at sample mean. Follow-up simple slopes analyses showed that the VR-CR increasing rate of change was statistically significant, $\gamma = 4.62$, se=1.43, $p = .002$. The slope for the VR-CG group was not statistically significant, $\gamma = .004$, se=1.53, $p = .998$.

S5. QLS Instrumental by Time and Condition

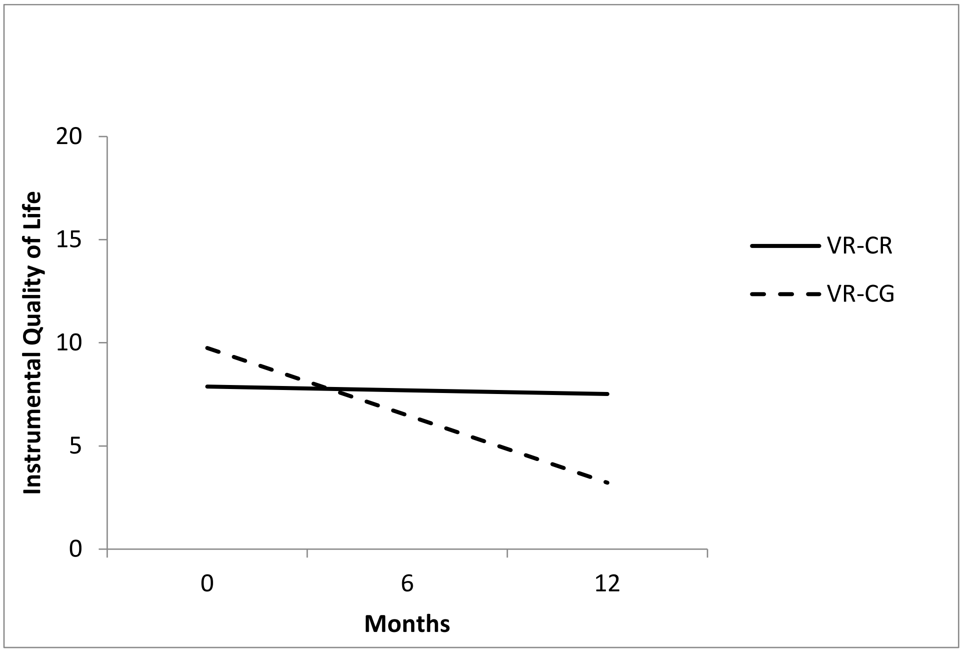

The rate of change in instrumental quality of life was significantly different in the Vocational Rehabilitation + Cognitive Remediation (VR-CR; solid lines) compared with the active control Cognitive Games condition (VR-CG; dashed lines). Training hours fixed at sample mean. Follow-up simple slopes analyses showed that the VR-CR rate of change was not statistically significant, γ = -.35, *se*=1.50, p = .815. The decreasing slope for the VR-CG group was highly statistically significant, γ = -6.53, *se*=1.67 $p < .001$.
